# Expression patterns of β-defensin and cathelicidin genes in parenchyma of bovine mammary gland infected with coagulase-positive or coagulase-negative *Staphylococci*

**DOI:** 10.1186/s12917-014-0246-z

**Published:** 2014-10-06

**Authors:** Ewa M Kościuczuk, Paweł Lisowski, Justyna Jarczak, Józef Krzyżewski, Lech Zwierzchowski, Emilia Bagnicka

**Affiliations:** Institute of Genetics and Animal Breeding, PAS in Jastrzębiec, ulPostępu 36A, Magdalenka, 05-552 Poland; Present address: Robert H. Lurie Comprehensive Cancer Center, Northwestern University, Chicago, IL USA

**Keywords:** Dairy cows, Udder parenchyma, Chronic mastitis, Antimicrobial peptides, Expression

## Abstract

**Background:**

Mastitis is still considered to be the most economically important infectious disease in dairy cattle breeding. The immune response in mammary gland tissues could help in developing support strategies to combat this disease. The role of neutrophils and macrophages in the innate response of mammary gland is well known. However, the immune response in mammary gland tissues, including levels of antimicrobial peptide transcripts, has not been well recognized. Moreover, most studies are conducted *in vitro*, on cell cultures, or on artificially infected animals, with analysis being done within a several dozen hours after infection.

The aim of the study was to examine the *in vivo* transcript levels of beta-defensin and cathelicidins genes in cow mammary gland secretory tissue (parenchyma) with the chronic, recurrent and incurable mammary gland inflammation induced by coagulase-positive or coagulase-negative *Staphyloccoci vs.* bacteria-free tissue*.*

**Results:**

The mRNA of *DEFB1*, *BNBD4*, *BNBD5*, *BNBD10* and *LAP* genes, but not of *TAP* gene, were detected in all investigated samples regardless of the animals’ age and microbiological status of the mammary gland, but at different levels. The expression of most of the beta-defensin genes was shown to be much higher in tissues derived from udders infected with bacteria (CoPS or CoNS) than from bacteria-free udders, regardless of parity. Cathelicidins (*CATH4*, *CATH5* and *CATH6*) showed expression patterns contrasting those of β-defensins, with the highest expression in tissues derived from bacteria-free udders.

**Conclusion:**

Increased expression of genes encoding β-defensins in the infected udder confirms their crucial role in the defense of the cow mammary gland against mastitis. On the other hand, the elevated cathelicidin transcripts in non-infected tissues indicate their role in the maintenance of healthy mammary tissues. The expression levels of investigated genes are likely to depend on the duration of the infection and type of bacteria.

**Electronic supplementary material:**

The online version of this article (doi:10.1186/s12917-014-0246-z) contains supplementary material, which is available to authorized users.

## Background

Mastitis is defined as chronic, latent or acute inflammation of the mammary gland, and is still considered to be the most economically important infectious disease in dairy cattle breeding [1]. The economic consequences of both clinical and sub-clinical mastitis include reduced milk yield, poor milk quality, increase in animal culling, and cost of veterinary services and medicine. The main pathogens causing mastitis in dairy cattle are contagious Gram-positive bacteria, such as *Staphylococcus aureus*, *Streptococcus agalactiae, Streptococcus dysgalactiae* and *Streptococcus uberis* [2-4]. The most common CoNS are *Staphylococcus chromogenes*, *Staphylococcus hyicus*, *Staphylococcus warneri*, *Staphylococcus epidermidis* and others [5]. Infection with major bacterial pathogens often leads to chronic asymptomatic inflammation, which persists throughout the animal’s life [6,7]. The contagious infection causes an increase in total somatic cell count (SCC) as a consequence of both leukocyte and epithelial cell numbers increasing, with or without clinical signs of mastitis. On the other hand, it is commonly believed that the environmental bacteria are less virulent than contagious ones, and are usually quickly eradicated by the host’s immune system [8,9]. However, opportunistic bacteria, such as coagulase-negative *Staphylococci* (CoNS) and *Escherichia coli* can also cause clinical and severe mastitis depending on cow factors (host defense status) [2-4]. The level of different antibacterial factors, such as lactoferrin, Natural killer cells, immunoglobulins, cytokines and many others rises dramatically during both clinical and sub-clinical inflammation [10,11]. β-Defensins and cathelicidins, among others, are the part of the antimicrobial arsenal of the leucocytes. As summarized by Bagnicka et al. [12] more than 20 β-defensins were found in cattle tissues, and several of them are expressed in the mammary gland. Until now, seven bovine cathelicidin genes with proved expression of peptides having the antimicrobial activity have been identified and some of them were found in milk from mastitic mammary glands [13]. However, Whelehan et al. [14] predicted the additional putative cathelicidin genes named *CATHL8*, *CATL2L2* and *CATHL9*. These three genes had the highest similarity to cathelicidin3 gene. Results of QTL mapping studies indicate β-defensin gene clusters as candidate regions influencing the number of somatic cells in milk [15,16]. In addition, the studies conducted by Ryniewicz et al. [17,18], Wojdak-Maksymiec et al., [19,20] and Bagnicka et al. [21,22] showed associations between polymorphisms in β-defensin genes and SCC, which could be used in genomic selection against mastitis.

Because resistance to mastitis has a genetic background, and genetic improvement is possible [15,23,24], the recognition of the immune response in mammary gland tissues could aid in developing support strategies to combat this disease. The role of neutrophils and macrophages in the innate immune response of mammary gland is well known [25]. However, the immune response in mammary gland tissues, including levels of antimicrobial peptide (AMP) transcripts, has not been well recognized [26]. Moreover, most studies are conducted *in vitro*, on cell cultures [27-31], or on artificially infected animals, with an analysis being done within several dozen hours after infection [32,33].

The Bovine Innate Immune Microarray, which comprised several defensin and cathelicidin genes among 1480 immune related genes, showed no differences in AMP genes expression upon the infection of the bovine mammary gland with *Staphylococcus aureus* [34]. Meta-analysis done by Genini et al. [35] reveled only cathelicidin antimicrobial peptide (CAMP) which increased upon mastitis infection.

Therefore, the aim of the present study was to estimate the *in vivo* transcript levels of β-defensin and cathelicidin genes in cow mammary gland secretory tissue (parenchyma) with the chronic, recurrent and incurable mammary gland inflammation caused by coagulase-positive or coagulase-negative *Staphyloccoci vs.* bacteria-free tissue.

## Results

### Health status of mammary gland

No bacteria were found in 18 samples of milk. In the remaining 47 samples only bacteria from the *Staphyloccocus* genus were stated; in 25 samples they were coagulase-positive (CoPS) and in 22 they were coagulase-negative (CoNS).

The highest average number of somatic cells (SC) and the lowest lactose content were found in milks containing coagulase-positive *Staphyloccoci* in lactations 3/4 (Table 1). For both types of infectious bacteria, as well as in groups with uninfected milk, the older animals (lactations 3/4) contained higher numbers of SC and lower levels of lactose than the younger ones (lactations 1/2).

**Table 1:** **The average number of somatic cells and lactose content in groups of animals**

**Group**	**N**	**Trait**			
		**SCC**		**Lactose [%]**	
		**Mean**	**SD**	**Mean**	**SD**
CoPS1/2	14	3052	2089	3.97	0.98
CoPS3/4	14	7014	3443	3.63	0.83
CoNS1/2	7	447	403	4.67	0.12
CoNS3/4	9	3924	3091	4.08	1.12
H1/2	9	47	20	4.50	0.26
H3/4	9	550	183	4.41	0.42

SCC – somatic cell count.

SD - Standard deviation.

CoPS1/2 - coagulase-positive Staphyloccoci infected cows in 1^st^ and 2^nd^ lactations.

CoPS3/4 - coagulase-positive Staphyloccoci infected cows in 3^rd^ and 4^th^ lactations.

CoNS1/2 - coagulase-negative Staphyloccoci infected cows in 1^st^ and 2^nd^ lactations.

CoNS1/2 - coagulase-negative Staphyloccoci infected cows in 3^rd^ and 4^th^ lactations.

H1/2 - pathogen-free cows in 1^st^ and 2^nd^ lactations.

H3/4 - pathogen-free cows in 3^rd^ and 4^th^ lactations.

### Expression of β-defensin genes

The transcripts of all studied β-defensin genes, except for *TAP* (tracheal microbial peptide) mRNA, were found in all investigated samples regardless of the animals’ age and microbiological status of the mammary gland, but at different levels. Two different pairs of primers previously used by Whelehan et al. [23] and Alva-Murillo et al. [29] to detect *TAP* mRNA in bovine mammary glands were used in the present study, but no expression of *TAP* mRNA was detected in the cows’ mammary gland parenchyma. Previously, these primers were used by us to amplify *TAP* sequences from the pooled bovine cDNA and gave the expected 151 bp and 216 bp amplification products.

**Figure 1:**
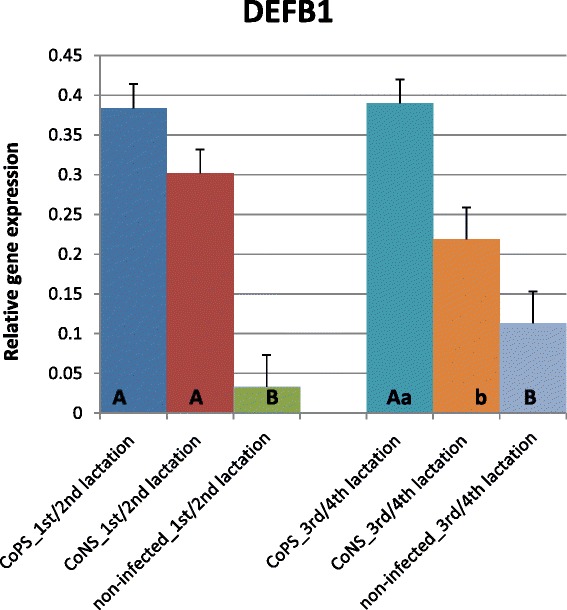
**Expression of β-defensin1 gene with its standard errors (SE).** *DEFB1* expression in secretory tissues of cow mammary gland infected with coagulase-positive or coagulase-negative *Staphylococci* compared to non-infected samples according to number of lactation; A,B – means differ within parities at p ≤ 0.01. a,b – means differ within parities at p ≤ 0.05

**Figure 2:**
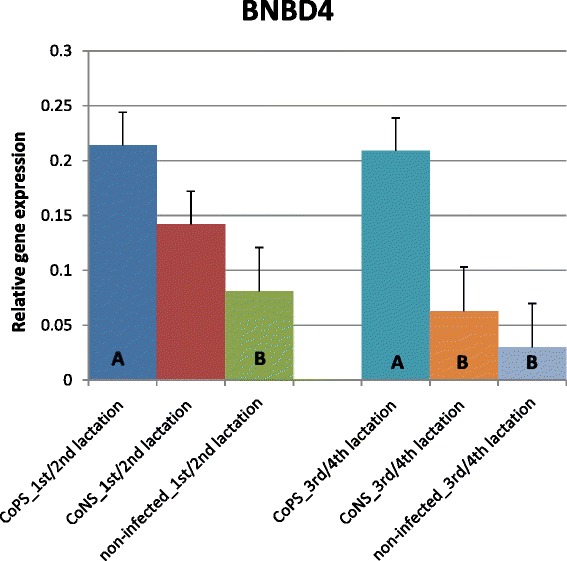
**Relative expression of β-defensin4 gene with its standard errors (SE).** Expression of *BNBS4* in secretory tissues of cow mammary gland infected with coagulase-positive or coagulase-negative *Staphylococci* compared to non-infected samples according to number of lactation; A,B – means differ within parities at p ≤ 0.01.

**Figure 3:**
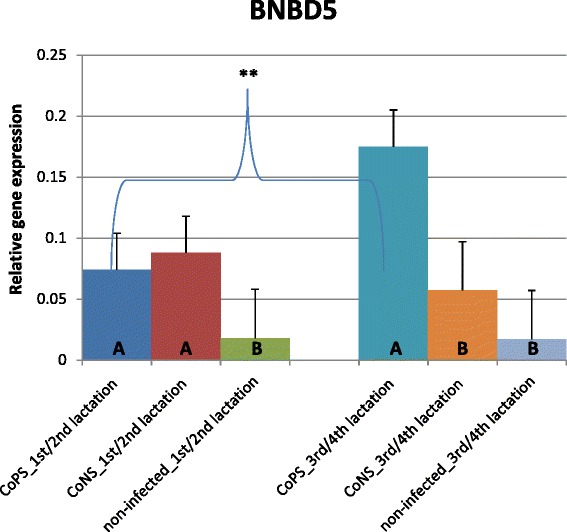
**Relative expression of β-defensin5 gene with its standard errors (SE).** Expression of BNBD5 in secretory tissues of cow mammary gland infected with coagulase-positive or coagulase-negative *Staphylococci* compared to non-infected samples according to number of lactation; A,B – means differ within parities at p ≤ 0.01. a,b – means differ within parities at p ≤ 0.05. **- means differ between parities at p ≤ 0.01

**Figure 4:**
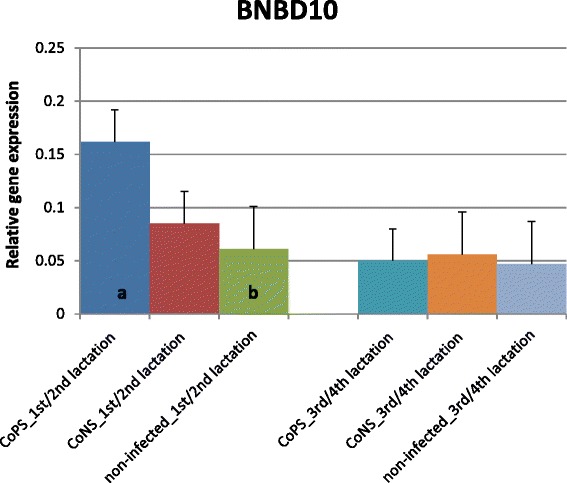
**Relative expression of β-defensin10 gene with its standard errors (SE).** Expression of *BNBD10* in secretory tissues of cow mammary gland infected with coagulase-positive or coagulase-negative *Staphylococci* compared to non-infected samples according to number of lactation; a,b – means differ within parities at p ≤ 0.05

**Figure 5:**
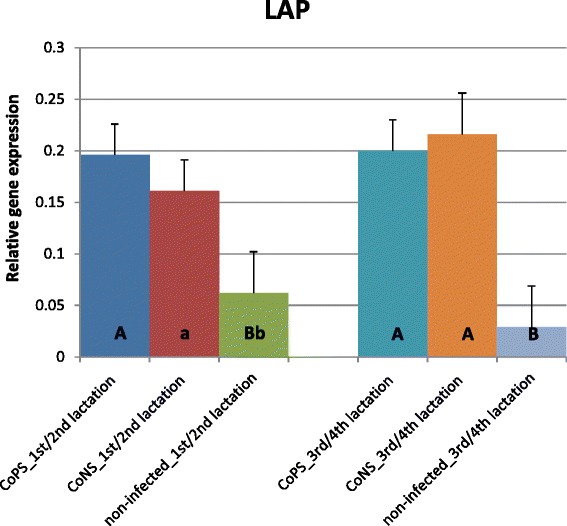
**Relative expression of lingual antimicrobial peptide gene with its standard errors (SE).** Expression of *LAP* in secretory tissues of cow mammary gland infected with coagulase-positive or coagulase-negative *Staphylococci* compared to non-infected samples according to number of lactation; A,B – means differ within parities at p ≤ 0.01. a,b – means differ within parities at p ≤ 0.05

The expression of most of the investigated β-defensin genes was shown to be much higher in tissues derived from quarters with stated bacteria in milk (CoPS or CoNS) than in tissues from bacteria-free udders, regardless of parity (Figures 1, 2, 3, 4 and 5). The differences between CoPS *vs*. CoNS infections were found due to the expression of *DEFB1*, *BNBD4* and *BNBD5* genes, and only in tissues derived from the mammary glands of animals in their lactations 3/4 (Figures 1, 2 and 3).

In lactations 1/2 the levels of *DEFB1*, *BNBD4* and *LAP* transcripts were ca. 12, 2.5 and 3-fold higher, respectively, in the CoPS infected tissues than in non-infected tissues (Figures 1, 2 and 5). The CoPS3/4-infected cows showed *DEFB1*, *BNBD4* and *LAP* expression levels 3.5, 7 and 7-fold higher, respectively, than healthy ones (Figures 1, 2 and 5). Thus, the expression levels were roughly the same in tissues derived from udders infected with CoPS, regardless of the parity.

When tissues infected with CoNS were compared to those derived from bacteria-free udders, the differences were revealed in the expression levels of *DEFB1* and *BNBD5* genes (Figures 1 and 4); in lactations 1/2 it was 9 and 5-fold higher, respectively. With cows in lactations 3/4 a 2.5-fold higher expression of *LAP* mRNA was stated in CoNS-infected *vs.* uninfected tissues.

Only a few differences were shown in the expression levels of defensin genes between age groups of cows. In the animals infected with CoPS, the expression of the *BNBD5* gene was twofold higher in tissues derived from the mammary glands of cows in lactations 3/4 than those that in lactations 1/2 (Figures 3 and 4). The opposite results were obtained for the *BNBD10* gene; expression was 3-fold higher in groups of animals in lactations 1/2 than in 3/4 (Figure 2). Moreover, the expression of the *DEFB1* gene was 3.5-fold higher in non-infected tissues derived from cows in lactations 3/4 than in 1/2 (Figure 1).

### Expression of cathelicidin genes

**Figure 6:**
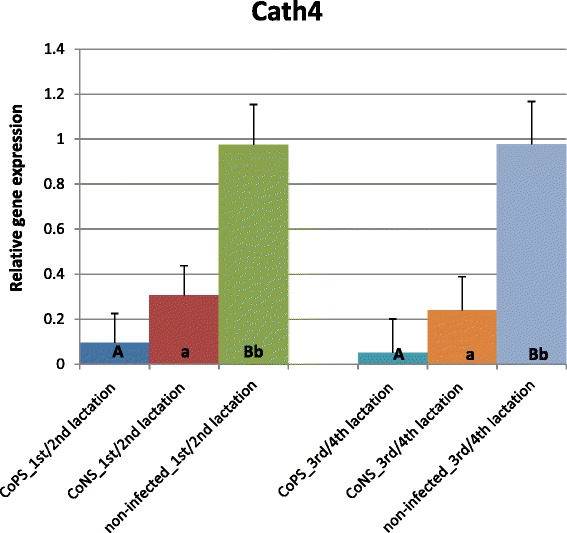
**Relative expression of cathelicidin4 gene with its standard errors (SE).** Expression of *CATH4* in secretory tissues of cow mammary gland infected with coagulase-positive or coagulase-negative *Staphylococci* compared to non-infected samples according to number of lactation; A,B – means differ within parities at p ≤ 0.01. a,b – means differ within parities at p ≤ 0.05.

**Figure 7:**
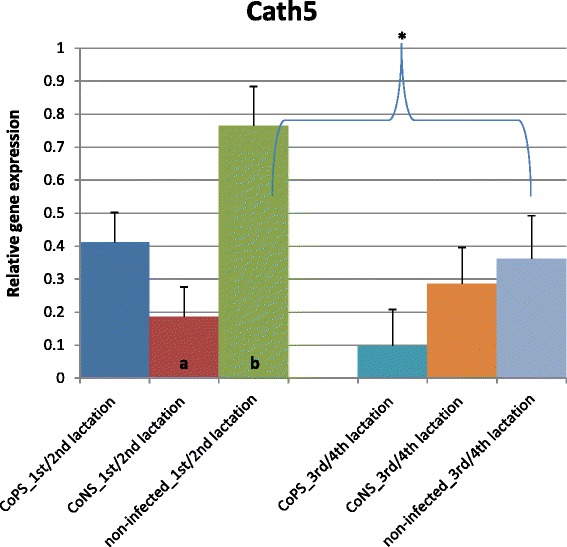
**Relative expression of cathelicidin5 gene with its standard errors (SE).** Expression of *CATH5* in secretory tissues of cow mammary gland infected with coagulase-positive or coagulase-negative *Staphylococci* compared to non-infected samples according to number of lactation; a,b – means differ within parities at p ≤ 0.05. *- means differ between parities at p ≤ 0.05.

**Figure 8:**
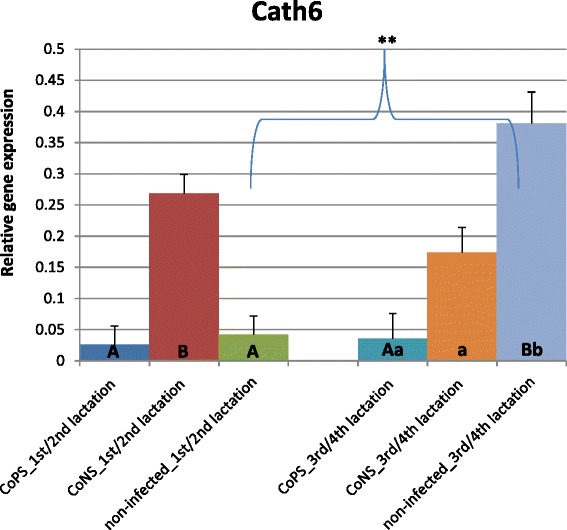
**Relative expression of cathelicidin6 gene with its standard errors (SE).** Expression of *CATH6* in secretory tissues of cow mammary gland infected with coagulase-positive or coagulase-negative *Staphylococci* compared to non-infected samples according to number of lactation; A,B – means differ within parities at p ≤ 0.01. a,b – means differ within parities at p ≤ 0.05. **- means differ between parities at p ≤ 0.01

Cathelicidins (*CATH*) showed expression patterns contrasting those of β-defensins (Figures 6, 7 and 8). For all *CATHs* the highest levels of mRNA were found in healthy tissues (except for *CATH6* expression in 1/2 lactation group). Moreover, the expressions levels of *CATH6* in both age groups were higher in tissues infected with CoNS than with CoPS (Figure 8). Differences between age groups were found only for the expression levels of *CATH5* and *CATH6* in bacteria-free tissues (Figure 7). The observed 3 and 4-fold differences between *CATH4* expression in tissues infected with CoNS *vs*. CoPS in both age groups were not statistically confirmed, possibly because of the high standard errors (SE) for LSMEAN solutions (Figure 6). In general, much higher standard errors were found for solutions obtained for expression results of cathelicidins’ than for defensins’ genes. This testifies to the higher variability between individuals of cathelicidin *vs*. defensin genes’ expressions.

## Discussion

The samples used in the current study were parenchyma samples, and were not homogenous secretory tissues. Parenchyma consists of alveoli that are lined with milk-secreting (inner epithelial cells) cuboidal cells and surrounded by myoepithelial cells. In the samples the lactiferous ducts lined by a stratified squamous keratinized epithelium and blood vessels also occurred. However, the secretory cells are the major component of parenchyma during lactation.

Until now there has been only limited information on β-defensin gene expression levels in parenchyma of mammary glands naturally infected with coagulase-positive or coagulase-negative *Staphylococci*. Most studies have focused on comparison of the cow mammary gland immune response to the bacterial infection caused by *E. coli* and *S. aureus*, which are the major pathogens in cows’ mastitis. Moreover, most these studies were conducted *in vitro* on culture of cells derived from the mammary parenchyma.

The results of the present study suggest that the expression of most β-defensin genes (except *TAP*) in parenchyma derived from healthy mammary glands is constitutive and on similar levels, regardless of gene and parity (Figures 1, 2, 3, 4 and 5). These findings confirmed the results of Tetens et al. [15], who reported a constitutive expression of *DEFB1, BNBD4*, *BNBD10* and *LAP*, and those of Goldammer et al. [2] who detected expression of *LAP*, *DEFB1*, *BNBD4*, *BNBD5*, and *BNBD10* in parenchyma from cow udders free from pathogens. It is noteworthy that Tetens et al. [15] also did not find the *TAP* gene transcripts in the mammary secretory tissues of healthy cows, which agrees with our results. However, in the study by Roosen et al. [36] *TAP* mRNA expression was detected in the healthy lactating parenchyma of the mammary gland, but only in one out of nine cows examined. The constitutive expression of β-defensin genes in other tissues of dairy cattle has also been proved. Until now, high expression of *BNBD4* and *BNBD5* genes was found in bovine alveolar macrophages, while the *DEFB1* (*EBD*) gene was highly expressed in the distal small intestine and colon, and also in healthy calves [37,38]. Moreover, *LAP* gene transcripts were identified in tongue and tracheal epithelial cells of healthy bovines [39,40].

Several studies confirmed both the constitutive and inducible expression of β-defensin genes in cows’ mammary gland alveoli. For example, Roosen et al. [36] identified *LAP* transcripts in both healthy and mastitis-affected mammary gland tissue. It has been proved that the expression level of β-defensin genes depends both on the presence and on the type of the infectious bacteria. In the present study expression of all studied β-defensin genes (except for *BNBD10*) was significantly higher in infected than in healthy tissues (Figures 1, 2, 3, 4, 5 and 6). Our results also showed that the expression levels of *DEFB1*, *BNBD4*, and *BNBD5* genes (Figures 1, 2 and 3) were higher in tissues infected with coagulase-positive *Staphylococci* (CoPS) than with coagulase-negative *Staphylococci* (CoNS), thus indicating that the organism’s reaction to infection may depend on the type of bacteria. However, in the case of *BNBD5* this difference was evident only in older cows, being in lactations 3 or 4. Previously, both *in vitro* and *in vivo* studies of the mammary secretory tissues have shown high expression of the *BNBD5* gene 84 h after infection with *S. aureus*, while in tissues infected with *E. coli* the reaction was almost immediate (after 24 h) [32,41,42].

Whelehan et al. [23] found higher *DEFB1, BNBD4* and *BNBD5* transcript levels in cows’ mammary gland parenchyma experimentally infected with *S. aureus* as compared to tissues derived from healthy udders. However, in contradiction to our results, they found no difference between *LAP* gene expression levels in infected *vs.* non-infected tissues – the discrepancy which could be explained by differences in the duration of infection; Whelehan et al. [23] measured the gene expression 30 days after infection, while our study focused on chronic and recurrent inflammation during lactation. However, the higher expression of *LAP* gene in CoPS- and CoNS-infected mammary parenchyma as compared to healthy tissue shown in the presented study confirmed the results of other authors [36,43], who also observed increased *LAP* expression in mastitis caused both by Gram-positive bacteria (*Streptococcus uberis*, *S. aureus*, and *Corynebacterium species*), and Gram-negative bacteria (*E. coli*). On the other hand, Günther et al. [27] pointed out that stimulated *LAP* gene expression did not correlate with the concentration of *E. coli* in the cultured mammary epithelial cells within 6 h after bacterial stimulation, because the relative copy number of *LAP* mRNA rose only ~25-fold over the control as compared to mRNA encoding immune effector serum amyloid A3 (SAA3), which increased 500-fold in the same period after stimulation. The highest differences in AMPs expression obtained in our study (in the naturally occurred, recurrent infections) were only ~12-fold between CoPS-infected and healthy tissues for β-defensin1, and ~19-fold between healthy and CoNS-infected tissues for cathelicidin4. Also noteworthy, the results obtained *in vivo* by Whelehan et al. [23] showed no more than a 27-fold change of *BNBD5* in alveoli after 30 days of the bacterial challenge. Fu et al. [44], within 24 h, found 10-fold and almost 100-fold higher *BNBD5* gene expression after the stimulation of the cultured epithelial cells derived from the mammary gland with *S. aureus* and *E. coli*, respectively. Under the same circumstances, the changes in *TLR2* and *TLR4* expression increased 2- and 8-fold after 3 h with the *S. aureus* and *E. coli* challenge, respectively. Furthermore, mRNA levels of cytokines, such as *TNF-α* (tumor necrosis factor α), *IL-8* (interleukin 8) and *IL-β* (interleukin β) increased only about 10-fold after *S. aureus* stimulation, while they increased 100-, almost 100-, and more than 1000-fold, respectively, within 3–6 h after the challenge with *E. coli*. However, the expressions of *IL-6* were less than 5-fold and only slightly higher than 10-fold after the *S. aureus* and *E. coli* challenge, respectively. These results suggest that the changes in AMPs expressions are not as high as those of some other immune proteins and peptides.

Our results also confirmed the findings of Tetens et al. [15], who observed that expression of *LAP* and *BNBD5* genes in the mammary tissues may be either constitutive or inducible in response to the *S. aureus* challenge. Moreover, Whelehan et al*.* [23] in *in vivo* study identified *DEFB1*, *BNBD4*, *BNBD5*, *LAP* and *TAP* transcripts in different parts of the mammary gland, including the alveoli, as a result of intramammary infection with *S. aureus.* Although the expressions of *BNBD4* and *BNBD5* were strongly induced, the expression of *DEFB1* was only moderately induced, while the expressions of *LAP* and *TAP* were not induced in mammary alveoli by the presence of bacteria. On the other hand, the study of Yang et al. [31], conducted in cell cultures derived from lactating cows’ alveoli, indicated significantly higher gene expression of both *BNBD5* and *TAP* after challenge with *S. aureus*. Furthermore, Fu et al. [44], in a study on cultured epithelial cells from bovine mammary glands also stated the up-regulation of *BNBD5* after stimulation with heat-inactivated *E. coli* or *S. aureus*. In our study, the expression of *TAP* was not found either in infected or healthy mammary tissues. Although it is difficult to explain such differences in *TAP* gene expression, the duration of inflammation might be one of the explanations. In earlier mentioned studies, the infections were artificially induced, and *TAP* expression was examined after 30 days (re-infused on day 28), but no later. Moreover, Petzl et al. [32] were not able to detect *BNBD5* gene transcript until 84-h after infection with *S. aureus*. In contrast, our study was conducted on naturally occurring, long-term and recurrent infections. Nevertheless, increased expression of most studied β-defensin genes in infected udder tissues obviously confirmed the participation of these AMPs in the defense of cow mammary gland against invasion by pathogenic bacteria.

So far, only several studies were conducted concerning on cathelicidin expression in mammary gland tissues. Tomasinsig et al. [33] found that 24 h after LPS treatment the expression levels of *CATH4* and *CATH6* were at the detection limit, while that of *CATH5* was the same as in non-treated tissues, both in mammary gland tissues and in cultures bovine epithelial cells. These results are consistent with ours, and also confirm the important role of the cathelicidins in healthy mammary tissues. In contrast, however, in the milk of cows, Smolenski et al. [45] stated that the cathelicidin peptide levels were increased only during induced mastitis. They explained this phenomenon through a rapid increasing of neutrophils in milk, which is the basic source of these peptides. They underlined that epithelial cells are not the main source of cathelicidins in milk during mastitis. These findings confirm our results – low expression of cathelicidins in parenchyma of infected udders. Moreover, Ibeagha-Awemu et al. [46] found the upregulated expression of *CATH14* in cultured mammary cells in response to *E. coli* and *S. aureus*. Smolenski et al. [45] obtained contradictory results in induced mastitis experiment *vs*. a naturally occurring infection. They found no cathelicidin expressions in mastitic milk samples (*in vivo*) collected in later stages of infection, probably because the apoptosis of neutrophils and domination of macrophages. These authors also hypothesize that infections with different bacteria caused different levels of cathelicidin expression. This finding was confirmed by the results of Whelehan et al. [14], who did not find any cathelicidin peptides in the milk of animals with naturally-occuring mastitis. In the alveolar region of the mammary gland (both healthy and experimentally-infected with *S. aureus* cows), however, they found the constitutive expression of *CATH5*. The expression of this cathelicidin was stable up to 48 hours after infection. Thus, the expression of some cathelicidin genes in mammary gland epithelium was proved in several studies, including ours. However, the new find of the current study was that their expression decreased in parenchyma obtained from mastitic animals.

The previous *in vitro* studies indicated a synergistic activity of defensins and cathelicidins [47]. However, *in vivo* the synergy of peptides from these two families may not occur, possibly due to of the different salt concentration in healthy and infected tissues. The inhibitory effect of the physiological salt concentration (150 mM NaCl), occurring in healthy tissues, on the activity of defensins as well as the resistance of cathelicidin genes in the physiological or even higher NaCl concentration were proved [47,48]. Therefore, cathelicidins may constitute the first line of defense in healthy tissues thanks to the retaining the activity at a physiological salt concentration. This phenomenon was confirmed by current study. Our results also suggest that the low salt concentration inhibits expression of cathelicidins genes and activates defensin expressions. The opposing results for defensin and cathelicidin genes shown in the current study might indicate on lack of synergy in activity *in vivo* because their activity depends on salt concentration in different way.

The *in vivo* studies known to the authors of presented publication did not report the age of examined cows, thus it not possible to compare our results with others. However, as we have shown, only the expression of *BNBD5*, *CATH5* and *CATH6* differs between age groups of animals within the healthy state of the mammary gland.

## Conclusion

Increased expression of genes encoding β-defensins in the infected udder confirms their crucial role in the defense of cow mammary glands against mastitis. On the other hand, the elevated cathelicidin transcripts in non-infected tissues indicate their role in the maintenance of healthy mammary tissues. The expression levels of investigated genes are likely to depend on the duration of the infection and type of bacteria.

## Methods

### Animals and tissue samples

The study was conducted on 40 Polish Holstein-Friesian dairy cows of Black and White variety. The animals were born and maintained in the Experimental Farm of the Institute of Genetics and Animal Breeding in Jastrzębiec, Poland. The animals were kept under identical conditions in a loose barn with free access to water. They were fed the same total mixed ration (TMR) diet *ad libitum*, consisting of corn silage (75%), concentrates (20%) and hay (5%), supplemented with a mineral and vitamin mixture, according to the INRA system [49]. The cows were between their first and fourth lactations. Animals were culled at the end of lactation (286 days, SD = 27) due to reproduction problems or chronic, usually asymptomatic, recurrent and incurable mammary gland inflammation. They were slaughtered in a registered slaughter house under conditions which are under constant monitoring by the institutional authorities. Immediately after the slaughter, the mammary tissue samples were taken from deep in the secretory part of the gland (parenchyma) from each quarter. They were rinsed in PBS to remove, as accurately as possible, the milk and blood from tissue samples, and frozen in liquid nitrogen (altogether 160 samples). The samples were stored at -80°C until further analysis.

All procedures involving animals were performed in accordance with the Guiding Principles for the Care and Use of Research Animals, and were approved by the III Local Ethics Commission (Warsaw University of Life Science; Permission No. 15/2010).

### Presence of bacteria, somatic cell count and lactose content in milk

Milk samples were taken from each quarter of the udder two days before slaughter and examined for the presence of bacteria. The milk was streaked on agar with 5% sheep blood (Columbia, bioMérieux, Craponne, France) and Chapman–Mannitol Salt Agar MSA (bioMérieux, Craponne, France) and incubated at 37°C for 18-24 h. Phenotypic evaluation of isolates included colony morphology, cell morphology and biochemical properties. Production of coagulase by *Staphylococci* was detected using a tube test with rabbit plasma. Additionally, *S. aureus* strains were identified using SlidexStaph-Kit (bioMérieux, Craponne, France).

### Design of the study

The tissue samples were divided into six groups according to the parity and health status of the mammary gland, which was established on the basis of the analysis of the microbiological status of the quarter milk, SCC, and lactose content. Previous statistical analysis (variance analysis with GLM procedure of SAS/STAT software) revealed no differences between the first and second as well as between the third and fourth lactations in AMP gene expression levels, and therefore the samples were grouped into two parity classes: lactations 1/2 and 3/4. The two control groups consisted of samples collected from bacteria-free mammary glands of cows in lactations 1/2 and 3/4 (H1/2 and H3/4, respectively). The experimental groups consisted of samples collected from cows infected with coagulase-positive *Staphylococci* in lactations 1/2 and 3/4 (CoPS1/2 and CoPS3/4, respectively), and groups created from samples collected from cows infected with coagulase-negative *Staphylococci* in lactations 1/2 and 3/4 (CoNS1/2 and CoNS3/4, respectively). The groups H1/2 and H3/4 consisted of 9 samples each, the groups CoPS1/2 and the group CoPS3/4 of 14 samples in each, while CoNS1/2 consisted of 7 and CoNS3/4 consisted of 9 samples, chosen on the basis of microbiological examination, out of 160 samples taken from the slaughtered cows. No more than two quarter samples were taken from each cow. The samples of the mammary gland infected with more than one type of bacteria were excluded from analysis.

### RNA isolation

Total RNA from the tissue samples was isolated using the RNeasy Mini Kit (Qiagen, Germany) with the DNAse digestion step according to the manufacture’s protocol. The quantity and quality of total RNA was estimated using NanoDrop (USA) and BioAnalyzer (Agilent, USA). Samples that meet the following criteria: >50 ng RNA with absorbance ratios A_260/280_ and A_260/230_ of ~2.0 and RIN (RNA Integrity Number) > 7.5, were used for further analyses.

RNA was reverse transcribed into cDNA using the Transcriptor First Strand cDNA Synthesis Kit (Roche, Switzerland). 0.5 μg RNA was firstly denatured at 65°C for 10 min in the presence of 50 μM oligo(dT). Next, reverse-transcription was conducted in a total volume of 20 μl of mixture composed of 13 μl of RNA, 4 μl of reverse transcriptase buffer, 2 μl of 10 mM deoxynucleotides, 0.5 μl of protector RNase Inhibitor (40U/μl), and 0.5 μl of reverse transcriptase (20U/μl). The mixture was incubated at 50°C for 60 min, then at 85°C for 5 min, and finally stored at 20°C.

### Quantitative Real Time PCR assays (qPCR)

Primers were designed with Primer 5 software (http://www.ncbi.nlm.nih.gov/tools/primer-blast/) according to the GenBank bovine sequences, or based on literature [23,29,33] (Table 2). Primers were designed only for those genes whose complete sequences are available in the GenBank. The transcript levels of the following defensins and cathelicidins were investigated in mammary gland parenchyma: β-defensin1 (enteric β-defensin), neutrophil β-defensin4, neutrophil β-defensin5, neutrophil β-defensin10, tracheal antimicrobial peptide (*TAP*), lingual antimicrobial peptide (*LAP*), cathelicidin4 (indolicidin), cathelicidin5 (bovine myeloid antimicrobial peptide28) and cathelicidin6 (bovine myeloid antimicrobial peptide27). The data for the reference genes contains an Additional file 1.

**Table 2:** **The primer sequences, amplicon length, melting temperature and no. of GenBank access of studied genes**

**Gene name**	**Gene symbol**	**Primersequence 5′-3′**	**Length of amplicon (bp)**	**Melting temp. (°C)**	**GenBank No access**
Cathelicidin 4 = indolicidin	CATHL4*	ACCCATCCAATGACCAGTTTGACC TTCACTGTCCAGAAGCCCGAATCT	177	60	X67340.1
Cathelicidin 5 = bovine myeloid antimicrobial peptide 28	CATHL5* = *BMAP28*	TCGGGAGTAACTTCGACATCACCT GGCCCACAATTCACCCAATTCTGA	141	60	X97609.1
Cathelicidin 6 = bovine myeloid antimicrobial peptide 27	CATHL6* = *BMAP27*	ATGGGCTGGTGAAGCAATGTGTAG TGGAGTAGCGGAATGACTGGAGAA	163	60	X97608.1
β-defensin1 = enteric β-defensin	*DEFB1* = *EBD*	ATCCTCTAAGCTGCCGTCT AGCATTTTACTGAGGGCGT	102	58	NM_175703.3
β-defensin4 = bovine neutrophil β-defensin4	*DEFB4 = BNBD4*	CGTTCTTGTGCCGTGTAG AAATTTTAGACGGTGTGTTG	149	58	NM_174775
β-defensin5 = bovine neutrophil β-defensin5	*DEFB5 = BNBD5*	TCCTCGTGCTCCTCTTCCTA CATATTCCAACGGCAGCTTT	143	58	NM_001130761
β-defensin10 = bovine neutrophil β-defensin10	*DEFB10 = BNBD10*	AGTTATCTAAGCTGCTGGG CGCTCTGTCAAAGGGTC	173	58	NM_001115084
Tracheal antimicrobial peptide	TAP**	TCCTGGTCCTGTCTGCTTC CTACAGCATTTTACTGCCCG	151	58	NM_174776
Tracheal antimicrobial peptide	TAP***	GCGCTCCTCTTCCTGGTCCTG GCACGTTCTGACTGGGCATTGA	216	57	NM_174776
Lingual antimicrobial peptide	*LAP*	GAAATTCTCAAAGCTGCCGTA TCCTCCTGCAGCATTTTACTT	194	58	NM_203435

*primer sequences according to Tomasinsiget al. (2010) [33].

**primer sequences according to Whelehan et al. (2011) [23].

***primer sequences according to Alva-Murillo et al. (2012) [29].

qPCR was performed in the Light Cycler 480 (Roche, Mannheim, Germany) using 96-well optical plates with the SYBR Green technique. A PCR mix was prepared to give the indicated end concentrations: 3 μl water, 1 μl forward primer (10 μM), 1 μl reverse primer (10 μM), 5 μl of cDNA, and SYBER Green I Master Mix 2× conc. 10 μl (Roche, Germany). The following amplification protocol was used: 5 min pre-incubation at 95°C, 35 cycles of 3-segment amplification with 15 s at 95°C for denaturation, 30 s at 58-60°C for annealing and 20 s at 72°C for elongation. All runs included a negative control (without cDNA). A dissociation stage was added to verify the presence of one gene-specific peak and the absence of primer-dimer peaks. A 10-fold dilution series of cDNA was included in each run to determine PCR efficiency by constructing a relative quantification standard curve.

### Statistical analysis

An expression level of defensins and cathelicidins genes was shown as the means of relative mRNA abundances with their standard errors (SE). All trait data were tested tfor normality of distribution, and somatic cell count (SSC) was transformed to natural logarithm values (SCS). Analysis of variance was conducted with the Tukey-Kramer test [50] to test the effects of the presence of bacterial pathogens on the relative expression of target genes. Effects such as parity and presence/lack of bacteria in milk were taken into consideration in the statistical model (Additional file 2):

$$ {\mathrm{y}}_{\mathrm{i}\mathrm{j}\mathrm{k}}=\upalpha + {\mathrm{B}}_{\mathrm{i}} + {\mathrm{P}}_{\mathrm{j}} + {\mathrm{B}\mathrm{P}}_{\mathrm{i}\mathrm{j}} + {\mathrm{e}}_{\mathrm{i}\mathrm{j}\mathrm{k}} $$

were:

$$ {\mathrm{y}}_{\mathrm{ijkl}}\hbox{--}\ \mathrm{trait}\ \mathrm{value}, $$

$$ \upalpha \hbox{--}\ \mathrm{overall}\ \mathrm{mean}, $$

$$ {\mathrm{B}}_{\mathrm{i}}\hbox{--}\ \mathrm{fixed}\ \mathrm{effect}\ \mathrm{of}\ \mathrm{i}\hbox{--} \mathrm{t}\mathrm{h}\ \mathrm{presence}/\mathrm{lack}\ \mathrm{of}\ \mathrm{bacteria}\ \left(\mathrm{j}=1,2,3\right), $$

$$ {\mathrm{P}}_{\mathrm{j}}\hbox{--}\ \mathrm{fixed}\ \mathrm{effect}\ \mathrm{of}\ \mathrm{j}\hbox{-} \mathrm{t}\mathrm{h}\ \mathrm{parity}\ \left(\mathrm{j}=1,2\right), $$

$$ {\mathrm{BP}}_{\mathrm{ij}}\hbox{--}\ \mathrm{fixed}\ \mathrm{effect}\ \mathrm{of}\ \mathrm{i}\mathrm{nteraction}\ \mathrm{of}\ \mathrm{i}\hbox{-} \mathrm{t}\mathrm{h}\ \mathrm{presence}/\mathrm{lack}\ \mathrm{of}\ \mathrm{bacteria}\ \mathrm{and}\ \mathrm{j}\hbox{-} \mathrm{t}\mathrm{h}\ \mathrm{parity}\ \left(\mathrm{i}\mathrm{j}=1,\dots, 6\right), $$

$$ {\mathrm{e}}_{\mathrm{ijkl}}\hbox{--}\ \mathrm{random}\ \mathrm{error}. $$

## Additional files

Additional file 1:
**Supplementary dataBMC.**
Additional file 2:
**Animal Research Reporting In Vivo Experiments.**

## Abbreviations

AMP: Antimicrobial peptide
*BNBD10*: Bovine neutrophil beta-defensin10
*BNBD4*: Bovine neutrophil beta-defensin4
*BNBD5*: Bovine neutrophil beta-defensin5
CAMP: Cathelicidin antimicrobial peptide
CATH: Cathelicidins
SD: Standard deviation
SE: Standard error
CoNS: Coagulase-negative *Staphylococci*
CoPS: Coagulase-positive *Staphylococci*
CP: Crossing point
SCC: Somatic cell count
*DEFB1*: β-defensin1
*EBD*: Enteric beta-defensin
HKG: Housekeeping gene
*HPRT1*: Hypoxanthine phosphoribosyltransferase1
*IL-8*: Interleukin 8
*IL-β*: Interleukinβ
*LAP*: Lingual antimicrobial peptide
NF: Normalization Factor
SAA3: Serum amyloid A3
*TAP*: Tracheal microbial peptide
*TBP*: TATA box-binding protein
TMR: Total mixed ration
*TNF-α*: Tumor necrosis factor α
