# Supplementary material for: Expression patterns of β-defensin and cathelicidin genes in parenchyma of bovine mammary gland infected with coagulase-positive or coagulase-negative Staphylococci
Source: BMC Vet Res. 2014 Oct 6;10:246. doi: 10.1186/s12917-014-0246-z (PMC4194403; doi:10.1186/s12917-014-0246-z)

***Quantitative Real Time PCR assays (qPCR)***

The two reference genes were selected most stably expressed in the present experimental design, belonging to different functional classes from six housekeeping genes (HKGS) commonly used as references, using previously described methodology [48] (Table 1). GeNorm software [1] was used to identify the most stable reference genes using the Crossing Point (CP) values and considering the qPCR efficiency (E). Amplification efficiency was calculated based on the slope of the standard curve using the formula E=10^(-1/slope)^. Ct and E values were entered into the geNorm applet, which then ranked the genes basing on M-values, where the gene with the most stable expression has the lowest M-value [2]. This measure is based on the principle that the expression ratio of 2 ideal control genes is identical in all samples, regardless of the experimental conditions. For the two HK genes with the lowest M-values the Normalization Factor (NF) was calculated to establish the relative expression of target genes. Relative mRNA expression of target genes was calculated based on HKG NFs and using the mathematical model for relative quantification in qPCR described by Pfaffl [3] (2001).

***Selection of most stable reference genes for qPCR***

The M-values of all putative reference genes were low and ranged between 0.6 and 0.3, and thus all of them met the criteria for proper references. However, the pair of genes: hypoxanthine phosphoribosyltransferase1 (*HPRT1*) and TATA box-binding protein (*TBP*) demonstrated the greatest stability expression in bovine mammary gland in the present experimental conditions, and therefore were selected as references. The M-values of examined genes and pair of genes were shown in Figure 1.

***References***

1. Lisowski P, Pierzchała M, Gościk J, Pareek ChS, Zwierzchowski L: **Evaluation of reference genes for studies of gene expression in the bovine liver, kidney, pituitary, and thyroid**. *J Appl Gene* 2008, **49**:367–372.
2. Vandesompele J, De Preter K, Pattyn F, Poppe B, Van Roy N, De Paepe A, Speleman F: **Accurate normalization of real-time quantitative RT-PCR data by geometric averaging of multiple internal control genes**. *Genome Biology* 2002, **3**, RESEARCH0034.
3. Pfaffl MW: **A new mathematical model for relative quantification in real time RT-PCR**. *Nucleic Acids Res* 2001, **29**: e45.

Table 1. Primer sequence and biological function of candidate reference genes.

| Gene name | Gene Symbol | Biological function | Primer sequence | Accession number from GenBank | Ampliconlength(bp) | Melting temp. (°C) |
| --- | --- | --- | --- | --- | --- | --- |
| Β-actin | *ACTB* | Cytoskeletal structural protein | GAGCGGGAAATCGTCCGTGAC  GTGTTGGCGTAGAGGTCCTTGC | NC_007326 | 278 | 60 |
| Glyceraldehyde-3P-dehydrogenase | *GAPDH* | Oxidoreductase in glucose metabolism | ACCACTTTGGCATCGTGGAG  GGGCCATCCACAGTCTTCTG | U85042 | 75 | 58 |
| Succinate dehydrogenase complex subunit A | *SDHA* | Catalyzes the oxidation of succinate | GCAGAACCTGATGCTTTGTG  CGTAGGAGAGCGTGTGCTT | NC_007318 | 185 | 60 |
| TATA box-binding protein | *TATABP* | Transcription factor | ACAACAGCCTCCCACCCTATGC  GTGGAGTCAGTCCTGTGCCGTAA | NM_001075742 | 111 | 60 |
| Zeta polypeptide | *YWHAZ* | Signal transduction by binding to phosphoserine-containing protein | GCATCCCACAGACTATTTCC  GCAAAGACAATGACAGACCA | NW_001493253 | 120 | 60 |
| Hypoxanthine phosphoribosyltransferase1 | *HPRT1* | Enzyme which plays a central role in the generation of purine nucleotides through the purine salvage pathway | TGCTGAGGATTTGGAGAAGG CAACAGGTCGGCAAAGAACT | NW_001501830 | 154 | 58 |

Figure 1. Expression stability (M) of candidate reference genes


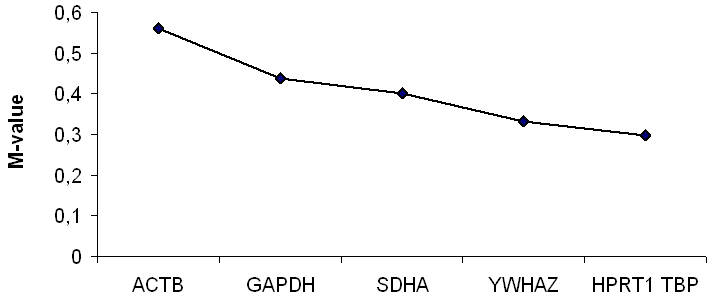

Supplement: Additional file 1: — Supplementary dataBMC. [file 12917_2014_246_MOESM1_ESM.docx]
