# Supplementary material for: Expression patterns of β-defensin and cathelicidin genes in parenchyma of bovine mammary gland infected with coagulase-positive or coagulase-negative Staphylococci
Source: BMC Vet Res. 2014 Oct 6;10:246. doi: 10.1186/s12917-014-0246-z (PMC4194403; doi:10.1186/s12917-014-0246-z)
Supplement: Additional file 2: — Animal Research Reporting In Vivo Experiments. [file 12917_2014_246_MOESM2_ESM.docx]

The ARRIVE guidelines

Animal Research: Reporting In Vivo Experiments:

Kościuczuk EM, Lisowski P, Jarczak J, Krzyżewski J, Zwierzchowski L, Bagnicka E: **Expression patterns of β-defensin and cathelicidin genes in parenchyma of bovine mammary gland infected with coagulase-positive or coagulase-negative *Staphylococci.***

Institute of Genetics and Animal Breeding, PAS, in Jastrzębiec, ul. Postępu 36A, 05-552 Magdalenka, Poland.

| ITEM | | Description |
| --- | --- | --- |
| Title | 1 | The beta-defensins and cathelicidins are the main families of antimicrobial peptides (AMPs). The transcription levels of several genes from both families were studied in cow udder parenchyma during mastitis. |
| Abstract | 2 | Mastitis is still considered to be the most economically important infectious disease in dairy cattle breeding. The immune response in mammary gland tissues, including levels of antimicrobial peptide transcripts, has not been well recognized. The transcription level of genes encoding beta-defensin and cathelicidins in cow mammary gland secretory tissue (parenchyma) with the chronic, recurrent and incurable mammary gland inflammation, induced by coagulase-positive or coagulase-negative *Staphyloccoci vs.* bacteria-free tissues were examined in vivo.  The study was conducted on 40 Polish H-F cows. The animals were culled at the end of lactation due to a chronic, recurrent and incurable mastitis. The mRNA levels of AMPs genes were estimated in mammary gland parenchyma infected with coagulase-negative or coagulase–positive *Staphyloccoci*. The control groups consisted of samples collected from bacteria free-mammary glands.  The expression of most of the investigated β-defensin genes was shown to be much higher in tissues derived from quarters with stated bacteria in milk (CoPS or CoNS) than in tissues from bacteria-free udders. Cathelicidins (*CATH*) showed expression patterns contrasting those of β-defensins (Fig 6-8). For all *CATHs* the highest levels of mRNA were found in healthy tissues (except for *CATH6* in expression 1/2 lactation group).  Increased expression of genes encoding β-defensins in the infected udder confirms their crucial role in the defense of cow mammary gland against mastitis. On the other hand, the elevated cathelicidin transcripts in non-infected tissues indicate their role in the maintenance of healthy mammary tissues. The expression levels of investigated genes are likely to depend on the duration of the infection and type of bacteria. |
| INTRODUCTION | | |
| Background | 3 | 1. Mastitis is defined as chronic, latent or acute inflammation of the mammary gland and is still considered to be the most economically important infectious disease in dairy cattle breeding [1]. β-Defensins and cathelicidins, among others, are the part of the antimicrobial arsenal of the leucocytes. Because resistance to mastitis has a genetic background and therefore the genetic improvement is possible [15, 23, 24], the recognition of the immune response in mammary gland tissues could aid in developing support strategies to combat this disease. The role of neutrophils and macrophages in the innate immune response of mammary gland is well known [25]. However, the immune response in mammary gland tissues, including levels of antimicrobial peptides (AMPs) transcripts, has not been well recognized [26]. Moreover, most studies are conducted *in vitro*, on cell cultures [27-31], or on artificially infected animals, with an analysis being done within several dozen hours after infection [32, 33]. 2. For this purpose we used 40 Polish Holstein-Friesian dairy cows naturally infected with *Staphyloccocusi* bacteria. Animals were culled at the end of lactation (286 days, SD=27) due to reproduction problems or chronic, usually asymptomatic, recurrent and incurable mammary gland inflammation. |
| Objectives | 4 | The aim of the study was to examine the *in vivo* transcript levels of beta-defensin and cathelicidins genes in cow mammary gland secretory tissue (parenchyma) with the chronic, recurrent and incurable mammary gland inflammation induced by coagulase-positive or coagulase-negative *Staphyloccoci vs.* bacteria-free tissue*.* |
| METHODS | | |
| Ethical statement | 5 | All procedures involving animals were performed in accordance with the Guiding Principles for the Care and Use of Research Animals, and were approved by the III Local Ethics Commission (Warsaw University of Life Science; Permission No. 15/2010). |
| Study design | 6 | 1. The tissue samples were divided into six groups according to the parity and health status of the mammary gland, which was established on the basis of the analysis of the microbiological status of the quarter milk, SCC, and lactose content. The two control groups consisted of samples collected from bacteria-free mammary glands of cows in lactations 1/2 and 3/4 (H1/2 and H3/4, respectively). 2. The experimental groups consisted of samples collected from cows infected with coagulase-positive *Staphylococci* in lactations 1/2 and 3/4 (CoPS1/2 and CoPS3/4, respectively), and groups created from samples collected from cows infected with coagulase-negative *Staphylococci* in lactations 1/2 and 3/4 (CoNS1/2 and CoNS3/4, respectively). 3. In the study N refers to the number of samples (samples taken from 40 slaughtered cows). 4. The animals were naturally infected with coagulase-negative or coagulase *Staphyloccoci*. The samples were taken during routine slaughter of culled cows in an officially registered slaughterhouse under conditions which are under constant monitoring by the institutional authorities. |
| Experimental procedures | 7 | Immediately after the slaughter, the mammary tissue samples were taken from deep in the secretory part of the gland (parenchyma) from each quarter. Total RNA from the tissue samples was isolated using the RNeasy Mini Kit (Qiagen, Germany) with the DNAse digestion step according to the manufacture’s protocol. RNA was reverse transcribed into cDNA using the Transcriptor First Strand cDNA Synthesis Kit (Roche, Switzerland). qPCR was performed in the Light Cycler 480 (Roche, Mannheim, Germany) using 96‑well optical plates with the SYBR Green technique. |
| Experimental animals | 8 | The study was conducted on 40 Polish Holstein-Friesian dairy cows of Black and White variety. The animals were born and maintained in the Experimental Farm of Institute of Genetics and Animal breeding in Jastrzębiec, Poland. |
| Housing and husbandry | 9 | The animals were kept under identical conditions in a loose barn with free access to water. They were fed the same total mixed ration (TMR) diet *ad libitum*, consisting of corn silage (75%), concentrates (20%) and hay (5%), supplemented with a mineral and vitamin mixture, according to the INRA system [49]. The cows were between their first and fourth lactations. |
| Sample size | 10 | The groups H1/2 and H3/4 consisted of 9 samples each, the groups CoPS1/2 and the group CoPS3/4 of 14 samples in each, while CoNS1/2 consisted of 7 and CoNS3/4 consisted of 9 samples, |
| Allocation animals to experimental groups | 11 | The groups of samples were created out of 160 samples by their choosing on the basis of microbiological examination. |
| Experimental outcomes | 12 | The primary outcome measures were the raw results of mRNA quantification of antimicrobial peptides (AMPs) from defensin and cathelicidins families based on qPCR.  The secondary outcome – was to asses correlations of AMPs mRNA levels with the type of bacterial infection and to assess the possible synergy of activity between peptides from two different families of AMPs (no synergy was found). |
| Statistical methods | 13 | An expression level of defensins and cathelicidins genes was shown as the means of relative mRNA abundances with their standard errors (SE) ranging between duplicates from 0.02 to 0.06. Thus, the results of real-time PCR measurements seem precise and reliable. All trait data were tested for normality of distribution, and somatic cell count (SSC) was transformed to natural logarithm values (SCS). Analysis of variance was conducted with the Tukey-Kramer test [50] to test the effects of the presence of bacterial pathogens on the relative expression of target genes. Effects such as parity and presence/lack of bacteria in milk were taken into consideration in the statistical model. |
| RESULTS | | |
| Baseline data | 14 | To estimate the animals’ health status the milk samples were taken from each quarter of the udder two days before slaughter and examined for the presence of bacteria. The milk was streaked on agar with 5% sheep blood (Columbia, bioMérieux, Craponne, France) and Chapman–Mannitol Salt Agar MSA (bioMérieux, Craponne, France) and incubated at 37°C for 18-24h. Phenotypic evaluation of isolates included colony morphology, cell morphology and biochemical properties. Production of coagulase by *Staphylococci* was detected using a tube test with rabbit plasma. Additionally, *S. aureus* strains were identified using SlidexStaph-Kit (bioMérieux, Craponne, France). |
| Number analyses | 15 | Group N  CoPS1/2 - 14  CoPS3/4 - 14  CoNS1/2 - 7  CoNS3/4 - 9  H1/2 - 9  H3/4 - 9  Out of 160 samples only 62 samples were taken to analysis. No more than two quarter samples were taken from each cow. The samples of the mammary gland infected with more than one type of bacteria were excluded from the analysis. The influence of infection of neighboring quarters on the gene expressions in the tissues of pathogen-free quarters was proved [23]. |
| Outcomes and estimation | 16 | The housekeeping genes (HKGs) used as qPCR references were selected carefully to current experiment. The M-values of all putative reference genes were low and ranged between 0.6 and 0.3, and thus all of them met the criteria for proper references. However, the pair of genes: hypoxanthine phosphoribosyltransferase1 (HPRT1) and TATA box-binding protein (TBP) demonstrated the greatest stability of expression in bovine mammary gland in the present experimental conditions, and therefore were selected as references.  All runs of qPCR analysis included a negative control (without cDNA). A dissociation stage was added to verify the presence of one gene-specific peak and the absence of primer-dimer peaks. A 10-fold dilution series of cDNA was included in each run to determine PCR efficiency by constructing a relative quantification standard curve. For each gene two runs were conducted and consistent results were obtained |
| Adverse events | 17 | The distribution of number of samples between groups was unequal and therefore the samples from 40 cows had to be taken to obtain sufficient numbers of samples in each group. |
| Interpretation/  science implications | 18 | The transcripts of all studied β-defensin genes, except for *TAP* (tracheal microbial peptide) mRNA, were found in all investigated samples regardless of the animals’ age and microbiological status of the mammary gland, but at different levels. No expression of *TAP* mRNA was detected in the cows’ mammary gland parenchyma. This could not be due to the inadequate primers or amplification procedure, since previously these primers were successfully used by us to amplify *TAP* sequences from the pooled bovine cDNA.  The expression of most of the investigated β-defensin genes was shown to be much higher in tissues derived from quarters with stated bacteria in milk (CoPS or CoNS) than in tissues from bacteria-free udders, regardless of parity. Cathelicidins (*CATH*) showed expression patterns contrasting those of β-defensins (Fig 6-8). For all *CATHs* the highest levels of mRNA were found in healthy tissues (except for *CATH6* in expression 1/2 lactation group). The results of the present study suggest that the expression of most β-defensin genes (except *TAP*) in parenchyma derived from healthy mammary glands is constitutive and on similar levels, regardless of gene and parity. Our results confirm the important role of the cathelicidins in healthy mammary tissues. The new findings of the current study show that the expression of cathelicidins decreased in parenchyma obtained from mastitic animals. Cathelicidins may constitute the first line of defense in healthy tissues thanks to the retaining the activity at a physiological salt concentration. The low salt concentration inhibits expression of cathelicidins genes and activates defensin expressions. The opposing results for defensin and cathelicidin genes shown in the current study might indicate on lack of synergy in activity *in vivo* because their activity depends on salt concentration in different way. |
| Generalisability/translation | 19 | Increased expression of genes encoding β-defensins in the infected udder confirms their crucial role in the defense of cow mammary glands against mastitis. On the other hand, the elevated cathelicidin transcripts in non-infected tissues indicate their role in the maintenance of healthy mammary tissues. The expression levels of investigated genes are likely to depend on the duration of the infection and type of bacteria. |
| Funding | 20 | Research was realized within the project “BIOFOOD – innovative, functional products of animal origin” no. POIG.01.01.02-014-090/09 co-financed by the European Union from the European Regional Development Fund within the Innovative Economy Operational Programme 2007 – 2013” and the National Science Centre of Poland No. NN311075339. |

Korhonen H, Marnila P, Gill HS: **Bovine milk antibodies for health**. *Br J Nutr* 2000, **1**:S135–146.

Tetens J, Friedrich JJ, Hartmann A, Schwerin M, Kalm E, Thaller G: **The spatial expression pattern of antimicrobial peptides across the healthy bovine udder.** *J Dairy Sci* 2010, **93**:775–783.

Whelehan, C. J, K. G. Meade, P. D. Eckersall, F. J. Young, and C. O'Farrelly. 2011. **Experimental Staphylococcus aureus infection of the mammary gland induces region-specific changes in innate immune gene expression**. *Vet. Immunol. Immunopathol*. **140**:181–189.

Sender G, Korwin-Kossakowska A, Pawlik A, Galal Abdel Hameed K, Oprządek J: **Genetic basis of mastitis resistance in dairy cattle – a review**. *Ann Anim Sci* 2013, **13**: 663–673.

Rainard P, Riollet C**: Innate immunity of the bovine mammary gland**. *Vet Res* 2006, **37**:369–400.

Brenaut P, Lefèvre L, Rau A, Laloë D, Pisoni G, Moroni P, Bevilacqua C, Martin P: **Contribution of mammary epithelial cells to the immune response during early stages of a bacterial infection to *Staphylococcus aureus***. Vet Res, 2014, **45** :16.

Günther J, Liu S, Esch K, Schuberth HJ, Seyfert H-M: **Stimulated expression of TNF-α and IL-8, but not of lingual antimicrobial peptide reflects the concentration of pathogens contacting bovine mammary epithelial cells**. *Vet Immunol Immunopathol* 2010, **135**:152–157.

Yang J, Sang Y, Meade KG, Ross CH: **The role of oct-1 in the regulation of tracheal antimicrobial peptide (TAP) and lingual antimicrobial peptide (LAP) expression in bovine mammary epithelial cells.** *Immunogenetics* 2011, **63**:715–725.

Alva-Murillo N, Ochoa-Zarzosa A, López-Meza JE: **Short chain Fatty acids (propionic and hexanoic) decrease *Staphyloccocusaureus* internalization into bovine mammary epithelial cells and modulate antimicrobial peptide expression**. *Vet Microbiol* 2012, **155**:324–331.

Téllez-Pérez AD, Alva-Murillo N, Ochoa-Zarzosa A, López-Meza JE: **Cholecalciferol (vitamin D) differentially regulates antimicrobial peptide expression in bovine mammary epithelial cells: Implications during *Staphyloccoccus aureus* internalization**. *Vet Microbiol* 2012, **160**:91–98.

Yang Z, Fu Y, Liu B, Zhou E, Liu Z, Song X, Li D, Zhang N: **Farrerol regulates antimicrobial peptide expression and reduces Staphylococcus aureus internalization into bovine mammary epithelial cells**. *Microb Pathogenesis* 2013, **65**:1–6.

Petzl W, Zerbe H, Günther J, Yang W, Seyfert HM, Nürnberg G, Schuberth HJ: ***Escherichia coli*, but not *Staphylococcus aureus* triggers an early increased expression of factors contributing to the innate immune defense in the udder of the cow**. *Vet Res* 2008, **39**:18 doi: 10.1051/vetres:2007057.

Tomasinsig L, De Conti G, Skerlavaj B, Piccinini R, Mazzilli M, D'Este F, Tossi A, Zanetti M: **Broad-spectrum activity against bacterial mastitis pathogens and activation of mammary epithelial cells support a protective role of neutrophil cathelicidins in bovine mastitis**. *Infect Immun* 2010, **78**:1781–1788.

Jarrige R. Éd., **Alimentation des bovines, ovins & caprins**. INRA Paris pp. 476, 1988.

SAS, SAS/STAT: **User’s Guide. Release 8e**. SAS Institute, Inc. Cary, NC, USA1999-2000.
